# Supplementary material for: A new model of decision processing in instrumental learning tasks
Source: eLife. 2021 Jan 27;10:e63055. doi: 10.7554/eLife.63055 (PMC7880686; doi:10.7554/eLife.63055)
Supplement: Transparent reporting form [file elife-63055-transrepform.docx]

***eLife’s* transparent reporting form**

We encourage authors to provide detailed information *within their submission* to facilitate the interpretation and replication of experiments. Authors can upload supporting documentation to indicate the use of appropriate reporting guidelines for health-related research (see [EQUATOR Network](http://www.equator-network.org/%20)), life science research (see the [BioSharing Information Resource](https://biosharing.org/)), or the [ARRIVE guidelines](http://www.plosbiology.org/article/info:doi/10.1371/journal.pbio.1000412) for reporting work involving animal research. Where applicable, authors should refer to any relevant reporting standards documents in this form.

If you have any questions, please consult our Journal Policies and/or contact us: [editorial@elifesciences.org](mailto:editorial@elifesciences.org).

**Sample-size estimation**

- You should state whether an appropriate sample size was computed when the study was being designed
- You should state the statistical method of sample size computation and any required assumptions
- If no explicit power analysis was used, you should describe how you decided what sample (replicate) size (number) to use

Please outline where this information can be found within the submission (e.g., sections or figure legends), or explain why this information doesn’t apply to your submission:

All data analyses consist of Bayesian estimation of parameters of cognitive models, for which statistical power (and corresponding sample size calculation) is ill-defined. Rather, the uncertainty related to sample size is directly reflected in the posterior distributions of interest, allowing for post-hoc assessment of the adequacy of sample size without violating any statistical assumptions as in the frequentist null-hypothesis testing framework (e.g., Wagenmakers, Psych Bull Rev. (2007), doi: https://doi.org/10.3758/BF03194105). Sample size was determined based on earlier literature using similar tasks and/or manipulations (e.g., Fontanesi et al., Psych Bull Rev. (2019), doi:10.3758/s13423-018-1554-2; Pedersen et al., Psych Bull Rev (2017) 24:1234–1251; Frank et al., J. Neuroscience (2015), doi:10.1523/JNEUROSCI.2036-14.2015; Van Maanen et al., J. Neuroscience (2011), doi:10.1523/JNEUROSCI.2924-11.2011), which are referenced in the main text.

Note further that for cognitive modelling contexts, trial numbers per subject is often considered more important than sample sizes (Smith & Little, Psych Bull Rev (2017), doi:10.3758/s13423-018-1451-8; Kolossa & Kopp, Neuroimage (2019), doi:10.1016/j.neuroimage.2018.01.005). Sufficiency of the amount of trials per participants was ensured by means of parameter recovery studies, which can be found in Figure 3-figure supplement 2, Figure 6-figure supplement 2, Figure 7-figure supplement 3, Figure 11-figure supplement 1.

**Replicates**

- You should report how often each experiment was performed
- You should include a definition of biological versus technical replication
- The data obtained should be provided and sufficient information should be provided to indicate the number of independent biological and/or technical replicates
- If you encountered any outliers, you should describe how these were handled
- Criteria for exclusion/inclusion of data should be clearly stated
- High-throughput sequence data should be uploaded before submission, with a private link for reviewers provided (these are available from both GEO and ArrayExpress)

Please outline where this information can be found within the submission (e.g., sections or figure legends), or explain why this information doesn’t apply to your submission:

Each experiment was performed once. Criteria for exclusion of data are defined in Methods, section “Exclusion” under experiment 1, experiment 2, and 4.

**Statistical reporting**

- Statistical analysis methods should be described and justified
- Raw data should be presented in figures whenever informative to do so (typically when N per group is less than 10)
- For each experiment, you should identify the statistical tests used, exact values of N, definitions of center, methods of multiple test correction, and dispersion and precision measures (e.g., mean, median, SD, SEM, confidence intervals; and, for the major substantive results, a measure of effect size (e.g., Pearson's r, Cohen's d)
- Report exact p-values wherever possible alongside the summary statistics and 95% confidence intervals. These should be reported for all key questions and not only when the p-value is less than 0.05.

Please outline where this information can be found within the submission (e.g., sections or figure legends), or explain why this information doesn’t apply to your submission:

The main statistical methods are Bayesian parameter estimation and model comparison, which are described in detail in the Methods section under *Bayesian hierarchical parameter estimation, posterior predictive distributions, model comparisons*. A brief overview of the methods is also presented in the main text under Results. Statistics describing the entire distributions of the posterior predictive distributions are presented throughout in figures 3-11. The mean and SD of the posterior parameter distributions of all estimated models can be found in Table 1.

(For large datasets, or papers with a very large number of statistical tests, you may upload a single table file with tests, Ns, etc., with reference to sections in the manuscript.)

**Group allocation**

- Indicate how samples were allocated into experimental groups (in the case of clinical studies, please specify allocation to treatment method); if randomization was used, please also state if restricted randomization was applied
- Indicate if masking was used during group allocation, data collection and/or data analysis

Please outline where this information can be found within the submission (e.g., sections or figure legends), or explain why this information doesn’t apply to your submission:

No experimental groups were used.

**Additional data files (“source data”)**

- We encourage you to upload relevant additional data files, such as numerical data that are represented as a graph in a figure, or as a summary table
- Where provided, these should be in the most useful format, and they can be uploaded as “Source data” files linked to a main figure or table
- Include model definition files including the full list of parameters used
- Include code used for data analysis (e.g., R, MatLab)
- Avoid stating that data files are “available upon request”

Please indicate the figures or tables for which source data files have been provided:

All data and the source code (R) used to analyze the data and generate the figures can be found on OSF (<https://osf.io/ygrve/>).
